# Common data elements collected among universities for sport-related concussion studies

**DOI:** 10.1186/s40621-018-0132-4

**Published:** 2018-02-12

**Authors:** Jingzhen Yang, Corinne Peek-Asa, James M. Noble, James Torner, Paul Schmidt, Martha L. Cooper, Big Ten – Ivy League Traumatic Brain Injury Research Collaboration Data Collection Working Group

**Affiliations:** 1 Department of Pediatrics, College of Medicine, Ohio State University, 700 Children’s Drive, WB5403, Columbus, OH 43205 USA; 2 Department of Occupational and Environmental Health, College of Public Health, University of Iowa, Iowa City, IA USA; 3 Department of Neurology, Taub Institute for Research on Alzheimer’s Disease and the Aging Brain, and G.H. Sergievsky Center, Columbia University, New York, NY USA; 4 Department of Epidemiology, College of Public Health, University of Iowa, Iowa City, IA USA; 5 Department of Athletics, University of Illinois, Champaign, IL USA; 6 Big Ten Academic Alliance, Champaign, IL USA; 7 Center for Injury Research and Policy, The Research Institute at Nationwide Children’s Hospital, Columbus, USA

**Keywords:** College sports, Common data element, Concussion

## Abstract

**Background:**

Universities are increasingly implementing programs to effectively respond to and manage sport-related concussions (SRCs). One such effort is to develop common data elements (CDEs) and standardize data collection methods. The objectives of this study were to describe CDEs currently collected by Big Ten and Ivy League universities for SRC studies, and to compare the data collected with the core CDEs recommended by the National Institute of Neurological Disorders and Stroke (NINDS).

**Methods:**

We conducted an anonymous cross-sectional online survey among medical staff at the 14 Big Ten and 8 Ivy League universities (one per university) between September and October 2015. The survey instrument, including 9 questions corresponding to the concussion data collected before, during, and after a concussion, was developed and pilot-tested before field use. We analyzed patterns of the concussion CDEs being collected, including when, what, and how the data were collected and stored, and compared them with the NINDS' recommended core CDEs.

**Results:**

A total of 19 out of 22 universities were included, with 13 from Big Ten and 6 from Ivy-League universities. All 19 participating universities currently collected concussion data with athletes before, during, and after a concussion. Great similarities in data collection were observed at baseline and acutely post-concussion across participating universities. All 19 universities collected at least one of the ten recommended acute symptoms checklists, and 18 universities collected one of the four recommended core neuropsychological function cognitive measures. However, CDEs in the sub-acute and chronic timeframes were limited, with only 9 (47%) universities collecting post-concussion short to long term outcome data. While over 60% of universities collected and stored concussion data electronically, only 17% to 42% of data collected were readily available for research.

**Conclusions:**

Significant inter-institutional similarities in acute concussion CDEs were found. Further efforts should focus on collecting sub-acute and chronic timeframe core CDEs and creating data access protocols to facilitate evidence-based concussion prevention and treatment for all collegiate athletes.

## Background

Concussions among collegiate athletes are a major public health and medical concern because of the high frequency and potential long-term consequences. College athletes have the highest risk for sport-related concussion (SRC) compared to other groups of athletes (Dompier, et al., 2015). Each year, an estimated 10,560 SRCs occur nationally among collegiate athletes participating in the 25 National Collegiate Athletic Association (NCAA) sports, with an overall concussion rate of 4.47 per 10,000 athlete-exposures (Wasserman et al. 2016; Zuckerman et al. 2015). Concussions can disrupt brain function, affecting athletes’ immediate physical, cognitive, emotional, and sleep health, and can also lead to other long-term, severe health conditions or threaten future athletic potential (Covassin et al. 2008; Guskiewicz et al. 2005; McCrea et al. 2003; Zuckerman et al. 2016). Despite increased public attention, the scientific understanding of effective SRC diagnosis, management and recovery among collegiate athletes remains limited, partly due to inconsistency in concussion data collection procedures and practices across universities and sports teams (Aukerman et al. 2016). Records of concussion-related information, including baseline, injury, post-concussion assessments and medical care, and academic accommodations may be spread across files kept by athletic departments, university student health systems, and other individual providers with or without direct affiliations with the teams or universities (Baugh et al. 2015; Baugh et al. 2016; Buckley et al. 2015). These data collection factors hinder the ability to study concussion incidence and outcomes (Maas 2009).

Comparisons across studies are equally challenging due to variability in data coding, definitions, data collection/storage procedures and assessment used in concussion studies and clinical practices coupled with the complexity and heterogeneity of concussion diagnosis (Aukerman et al. 2016; Buckley et al. 2015; Maas 2009). To allow data aggregation into significant metadata results so that comparisons across studies can be made in meaningful ways, it is important to understand different data definitions and data collection procedures. Identifying common data elements (CDEs) is a critical first step in data aggregation, which could help facilitate standardization of definitions, data elements and protocol inventories, and subsequent research data sharing, ultimately leading to a stronger evidence base for prevention, diagnosis and treatment of concussions (Adelson et al. 2012; Maas et al. 2011; McCauley et al. 2012).

Increased efforts have been made in recent years by scientific communities to develop CDEs for traumatic brain injury (TBIs) (Maas 2009; McCauley et al. 2012; Stone 2010; Wilde et al. 2010). In June 2017, the first set of CDEs for SRCs recommended by the National Institute of Neurological Disorders and Stroke (NINDS) was released, which classified CDEs as Core, Supplemental - Highly Recommended, Supplemental, or Exploratory and organized them based on post-injury timeframe of Acute (time of injury until 72 h), Sub-acute (after 72 h to 3 months), or Persistent/Chronic (3 months and greater post-concussion) (NINDS 2017). Since athletes with concussions generally report milder and more heterogeneous symptoms than athletes with more severe TBIs, the nuances of diagnosis and assessment are challenging with standard definitions and measures, making such standardization even more critical (Baugh et al. 2016; McCrea et al. 2013). One of the efforts to improve data comparisons is The Big Ten-Ivy League Traumatic Brain Injury Research Collaboration, a multi-institutional, collaborative research effort among physicians, researchers, and athletic trainers. One of the goals of this collaboration is to seek a better understanding of the causes and effects of SRCs on collegiate athletes though the coordination and integration of surveillance and research data collected by athletic and scientific investigators. The aims of the study were to describe concussion data elements currently collected by the Big Ten and Ivy League universities for SRC studies, including when, what, and how the data were collected and stored, and to compare the concussion data collected with the NINDS recommended core CDEs. The results of this study will help develop CDEs and standardize data collection methods for SRC research among collegiate athletes.

## Methods

### Study design and participants

We conducted a cross-sectional, confidential online survey via Survey Monkey between September and October 2015. We invited Head Athletic Trainers (ATs) or Directors of Sports Medicine at the 14 Big Ten universities and 8 Ivy League universities (*n* = 22) who were listed as being a part of The Big Ten-Ivy League Traumatic Brain Injury Research Collaboration to participate in this study. We sent a link to the online survey, along with a 3-digit code, via email to each contact person at the 22 universities. These individuals or their appropriate designees (e.g., team physicians or researchers affiliated with athletics who were members of within university SRC collaboration and highly qualified to respond on behalf of their institutions) completed the survey.

### Survey instrument

The survey instrument was developed by the Data Collection Working Group based on current concussion literature. We defined a concussion as a traumatically induced, transient disturbance of brain function involving a complex pathophysiological process in this study (Harmon et al. 2013). Collaboration experts reviewed the instrument, which was then pilot tested among head athletic trainers and TBI researchers, and revised based on the feedback received. The survey contained 9 questions corresponding to the 9 domains of concussion data collected as part of medical care performed by athletic departments before, during, and after a concussion. Specifically, three questions asked about concussion data collected at baseline pre-injury (demographics/pre-morbidity, history of concussion, and baseline testing), five questions asked about concussion data collected at the time when a concussion was suspected or acutely post-concussion (time/place/mechanism of injury, sideline assessment, concussion symptoms, neuropsychological tests, and postural stability tests), and one question asked about data collected on short to long term outcomes after athletes return to play (functional outcome and academic outcome). For each question, the participants were asked to respond on whether the data element(s) was collected, the assessments used for collecting the data (e.g., tools used to measure concussion symptoms), how the assessments were conducted (e.g., person(s) who collected data, and format of data collected and stored), and whether the data collected were readily available for concussion research. The survey contained no questions about individual athletes or concussion frequency.

### Data collection

Completion of the survey took about 10–15 min. By completing the survey, the respondent was consenting to participate. Respondents who were blinded to the researchers were ensured that all responses provided would remain confidential, and neither the individual participant nor the university would be identified in any report or publication. The survey allowed respondents to skip questions if they did not want to answer, and respondents were informed that they could end participation at any time.

A total of 20 out of 22 eligible universities participated in the online survey, with a response rate of 91%. Of these, we included 19 useable surveys in the analysis (excluding one survey with over 70% of responses missing), with 13 from Big Ten universities and 6 from Ivy-League universities.

### Data analysis

We transferred data collected from the online surveys to SAS version 9.3 for data analysis. We described the characteristics of the CDEs being collected and stored, and compared the data collected with the recommended core CDEs. We also assessed the differences in concussion data collection patterns between Big Ten and Ivy League universities and between universities that responded that their data were or were not available for research, using chi-square tests or Fisher’s exact tests, as appropriate. The statistical significant level was set at α = .05.

## Results

### Characteristics of concussion data collected

Of 19 universities included, all currently had a policy addressing SRC management. We observed a high rate of data collection by 19 participating universities for 8 out of 9 domains studied, ranging from 90% to 100% (Table 1). However, only 9 (47%) universities collected data regarding post-concussion short to long term outcomes. We did not find any statistically significant differences in current concussion data collection patterns (e.g., what was collected, instruments and methods used, or how data were stored) between Big Ten and Ivy League universities, or between universities that responded their data “could” and “could not” be made available for research.

**Table 1:** Concussion data collected before, during and after injury by Big Ten and Ivy-League universities

Domains of concussion data		n	(%)
Concussion data collected prior to injury			
1	Does your university collect any demographic and pre-morbid information about players at the beginning of their athletic season?	17	89.5
2	Does your university collect any concussion history information?	19	100.0
3	Does your university collect any baseline testing/information prior to when a concussion is suspected with an athlete?	19	100.0
Concussion data collected at injury and acutely post-injury			
4	Does your university currently collect any concussion information/data when a concussion is suspected with an athlete?	19	100.0
5	Does your university collect any information on sideline assessment at the time of a suspected concussion with an athlete?	18	94.7
6	Does your university collect information on athletes’ post-concussion symptoms?	18	94.7
7	Does your university collect any information from post-concussion neuropsychological tests?	19	100.0
8	Does your university collect any information from post-concussion postural stability tests?	17	89.5
Follow-up data collected after concussion resolution			
9	Does your university collect any information on post-concussion outcomes after a concussion resolution?	9	47.4

### Concussion data collected prior to injury

Data collection prior to concussion, including baseline testing, concussion history, and athletes’ demographic information, was implemented among most participating universities. All 19 participating universities conducted baseline testing prior to concussion. The most common tests conducted at baseline included postural stability test(s) (*n* = 19), neuropsychological test(s) (*n* = 18, 95%), and symptom information (*n* = 17, 90%). All 19 universities collected concussion history by asking the number of concussions an athlete had ever had. Seventeen universities (90%) also collected details regarding the most recent concussion, and 11 universities (58%) asked about the number of concussions in the past 12 months. The most commonly collected demographic information included sports, age, and gender, which were collected by 17 (90%) universities. Over two-thirds of participating universities also collected data on mental health status at baseline, including depression (*n* = 15, 79%), anxiety (*n* = 14, 74%), and ADHD (n = 17, 90%).

### Concussion data collected acutely post-injury

**Fig. 1:**
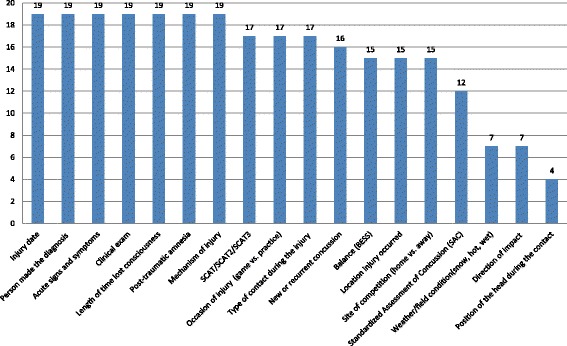
Data collected when a concussion is suspected with an athlete

When a concussion was suspected, all 19 participating universities collected injury information, 18 (95%) collected data from a sideline assessment, 18 (95%) collected data on post-concussive symptoms, and 19 (100%) conducted post-concussion neuropsychological function tests (Table 1). When a concussion was suspected, all 19 universities collected data on injury date, person who made the diagnosis, acute signs and symptoms, clinical exam measures, length of loss of consciousness (LOC), length of post-traumatic amnesia, and injury mechanism. Other common injury data collected included occasion of injury (game vs practice), type of contact during the injury, concussion type (new vs. recurrent), and location of injury (Fig. 1).

The three commonly used tools for sideline assessment were acute signs and symptoms, clinical exam, and Sport Concussion Assessment Tool 3 (SCAT-3) (Guskiewicz et al. 2013). For data collection on post-concussion symptoms, the instruments were varied and included the Graded Symptom Checklist (Janusz et al. 2012) (*n* = 9), Postconcussion Symptom Scale (PCS-S) (Lovell and Collins 1998) (*n* = 6) or Concussion Symptom Inventory (PCSI) (Randolph et al. 2009) (*n* = 6). Three universities used both the (PCS-S) and Graded Symptom Checklist. Thirteen universities recorded post-concussion symptoms daily until cleared for return to play, while other universities started with daily and then as needed per their study protocol or per direction of treating physicians.

### Concussion data collected after athletes return to play

Only 9 (47%) universities collected post-concussion outcomes after athletes’ return to play (Table 1). The follow-up window ranged from one week (*n* = 4), to 8 to 30 days (*n* = 3), to more than one month (*n* = 2). None of the universities followed concussed athletes longer than 6 months. Four universities collected functional outcomes, four collected academic outcomes, four collected sports performance outcomes, and two collected mental health outcomes.

### Methods used for concussion data collection and data storage

Most participating universities used an electronic format to collect and store concussion data in at least one domain (Table 2). In 2015, over 60% of universities collected and stored all 9 domains of concussion data either in all electronic records or both paper and electronically (e.g., collected on paper and stored electronically). In particular, more than half of universities collected and stored players’ demographics, concussion history, post-concussion neuropsychological function, and post-concussion outcomes in all electronic format. Athletic trainers collected most concussion data at baseline, or at sideline or acutely post-injury when an injury occurred. Athletes, on the other hand, completed most of the demographic information and concussion history information, as well as pre- and post-injury neuropsychological function tests. Physicians were also involved in data collection at injury, sideline, and post-concussion when medical care was needed. However, in any of the 9 domains, less than half (ranging from 17% to 42%) of universities indicated that concussion data they collected were readily available for research.

**Table 2:** Methods used in concussion data collection and storage by Big Ten and Ivy-League universities

		Universities Completed Data Collection	Method for Data Collection and Storage						Person Who Collected/Reported Data					
			All electronic data in 2015		Paper and electronic data in 2015		Available for research		Athletic trainer		Athlete		Physician	
		n	n	%	n	%	n	%	n	%	n	%	n	%
Concussion data collected prior to injury														
1	Does your university collect any demographic and pre-morbid information about players at the beginning of their athletic season?	17	10	58.8	7	41.2	6	35.3	9	52.9	16	94.1	8	47.1
2	Does your university collect any concussion history information?	19	10	52.652.6	8	42.1	6	31.6	11	57.9	17	89.5	8	42.1
3	Does your university collect any baseline testing/information prior to when a concussion is suspected with an athlete?	19	7	36.8	8	42.1	6	31.6	18	94.7	9	47.4	4	21.1
Concussion data collected at injury and acutely post-injury														
4	Does your university currently collect any concussion information/data when a concussion is suspected with an athlete?	19	8	42.1	10	52.6	8	42.1	18	94.7	6	31.6	12	63.2
5	Does your university collect any information on sideline assessment at the time of a suspected concussion with an athlete?	18	3	16.7	8	44.4	3	16.7	18	100.0	NA		11	61.1
6	Does your university collect information on athletes’ post-concussion symptoms?	18	6	33.3	5	27.8	3	16.7	18	100.0	8	44.4	16	88.9
7	Does your university collect any information from post-concussion neuropsychological tests?	19	12	63.2	4	22.4	6	33.3	NA		19	100.0	NA	
8	Does your university collect any information from post-concussion postural stability tests?	17	6	35.3	7	41.2	3	17.6	17	100.0	NA		7	41.2
Follow-up data collected after concussion resolution														
9	Does your university collect any information on post-concussion outcomes after a concussion resolution?	9	5	55.6	3	33.3	2	22.2	7	77.8	6	66.7	6	66.7

### Comparison of concussion data collected with the NINDS recommended core CDEs

Overall, acute concussion data collected by the participating universities were consistent with the NINDS recommended acute core CDEs (Table 3). Specifically, of 19 universities that collected neuropsychological function, 18 universities were consistent with one of the recommended four core neuropsychological function cognitive measures (17 used the ImPACT test (Lovell et al. 2000), and one used Axon Sports Computerized Cognitive Assessment Tool (CCAT) (CogSport, 1999). Seventeen universities assessed the recommended post-concussion Balance Error Scoring System (BESS) (Riemann et al. 1999) acutely (time of injury until 72 h). All 19 universities collected one of ten recommended acute core symptoms checklists, with most using SCAT-3 (*n* = 17), ImPACT (n = 17), SAC (*n* = 12), and/or PCSI (*n* = 9). No university in this study used Automated Neuropsychological Assessment Metrics (ANAM) (Cernich et al. 2007), CNS Vital Signs (Gualtieri & Johnson 2006), or The Rivermead Postconcussion Symptoms Questionnaire (RPQ) (King et al. 1995). Further, recommended concussion core CDEs at the sub-acute or persistent/chronic timeframes were not collected in the survey. Nevertheless, 10 out of 19 universities in this study did not collect follow-up data after concussed athletes returned to play.

**Table 3:** Core CDEs used for sports-related concussion studies by Big Ten and Ivy-League universities

Core CDEs recommended by the National Institute of Neurological Disorders and Stroke (NINDS)		CDEs used by Big Ten and Ivy-League universities	
Item	Instrument	Yes	No
		N (%)	N(%)
1	Balance Error Scoring System(BESS)	17 (89.5)	2 (10.5)
2	Sport Concussion Assessment Tool (SCAT-3) or −5	17 (89.5)	2 (10.5)
3	Standardized Assessment of Concussion (SAC)	12 (63.2)	7 (36.8)
4	Post-concussion Symptom Inventory (PCSI)	9 (47.4)	10 (52.6)
5	Child Sport Concussion Assessment Tool (Child-SCAT)	unknown^a^	
6	Immediate Post-Concussion Assessment and Cognitive Testing (ImPACT)	17 (89.5)	2 (10.5)
7	Post Concussion Symptoms Scale (PCS-S)	6 (31.6)	13 (68.4)
8	Axon Sports Computerized Cognitive Assessment Tool (CCAT)	1 (5.3)	18 (94.7)
9	Automated Neuropsychological Assessment Metrics (ANAM)	0	19 (100.0)
10	CNS Vital Signs	0	19 (100.0)
11	The Rivermead Postconcussive Symptom Questionnaire (RPQ)	0	19 (100.0)
Recommendation 1	Collect one of four core neuropsychological function cognitive measures (items #6, #8, #9,#10)	18 (94.7)	1 (5.3)
Recommendation 2	Collect BESS (item #1) acutely (< 72 h of post-injury)	17 (89.5)	2 (10.5)
Recommendation 3	Collect one of ten symptom checklists (Items #2 to #11) acutely (< 72 h of post-injury)	19 (100.0)	0
Recommendation 4	Collect one of five symptom checklists (Items #6 to #10) sub-acutely (72 h to 3 months post-injury)	unknown^a^	
Recommendation 5	Collect one of six symptom checklists (Items #6 to #11) in chronic timeframe (> 3 months post-injury)	unknown^a^	

^a^Information was not collected in this study

## Discussion

This study identified concussion CDEs currently collected among NCAA athletes at 19 universities in the Big Ten and Ivy League conferences and compared those with the NINDS recommended core CDEs. The main results showed that all 19 universities were currently collecting concussion data with athletes before, during, and after a concussion, with acute data collection being more consistent with the recommended symptoms checklist. There were great similarities in the concussion data currently being collected at baseline and acutely post-concussion across Big Ten and Ivy League universities, irrespective of whether the data collected were available for research. However, there was lack of short- and long-term follow-up data collection and CDEs in the sub-acute or persistent/chronic timeframes. Additionally, while over 60% of concussion data were collected and stored electronically, only 17% to 42% of data collected were readily available for research, possibly due to the combination of low data storage capacity and lack of consent by the participants. Improving current concussion data collection and storage could be useful in identifying potential support services for injured athletes and beneficial for concussion research (Broglio et al. 2014).

This is the first study to document current SRC CDEs collected among two, large Division I conferences. The results of this study provide empirical evidence that can potentially be used to arrive at consensus on the conceptual domains most relevant to SRC studies, and on the measurement tools that are most useful in quantifying those domains among collegiate athletes (Aukerman et al. 2016; Baugh and Kroshus 2016; Grinnon et al. 2012; Wilde et al. 2010). The results of this study also have important implications for future studies on effective concussion management not only for athletes in NCAA Division I conference universities, but also for athletes from small universities including Division 2 and 3 universities, where resources for concussion data collection may be less available.

Our results that all universities closely monitored acute concussion symptoms and impairments by collecting the recommended acute core CDEs likely reflect current established guiding principles for concussion diagnosis and management through the NCAA (Buckley et al. 2015; NCCA 2017; Putukian 2011). Since 2010, following enactment of its Concussion Policy and Legislation (Baugh et al. 2015; Buckley et al. 2015), the NCAA required each of its member schools to have a concussion management plan. More recently, the NCAA has taken additional steps to address concussion diagnosis and management in college athletes (Baugh and Kroshus 2016; Baugh et al. 2016; Broglio et al. 2014). For example, a well-developed pre-participation baseline concussion assessment is required for all varsity student athletes, which includes, but is not necessarily limited to a brain injury/concussion history, symptom evaluation, cognitive assessment, and balance evaluation (Kerr et al. 2015; NCAA 2017). Since 2013, the Ivy-League conference has required reporting of all concussion data to a central study. These requirements could theoretically have influenced data collection practices among participating universities. Additionally, all participating universities were from NCAA Division I universities and had one or more designated full-time, certified AT and a designated physician for each team. These individuals were present during the team practices and competitions, and work very closely with injured athletes from their initial injury to their return to unrestricted activity to ensure speedy and complete injury resolution and safe return to play. However, soon after concussed athletes return to play, medical care is often discontinued (Baugh et al. 2016; Kerr et al. 2015), as revealed in our results that less than half of universities collect follow-up data after athletes return to play. Our results, along with those of others (Maas 2009; McCauley et al. 2012; McCrea et al. 2013), call for further efforts to prospectively follow-up with concussed athletes and collect the recommended core sub-acute and chronic CDEs, to assess the short- and long-term outcome data after a concussion including academic outcomes. These data could promote continued care to injured athletes, even after they graduate or leave school as recently voted on by 65 schools in the five NCAA conferences (NCAA, 2018). These data could also help address the knowledge shortcomings regarding the long-term effects of concussions on the health and wellbeing of collegiate athletes (McCrea et al. 2013).

Despite the fact that all universities collected CDEs acutely post-concussion as recommended, the instruments or assessment tools used remain varied, which could hamper comparisons of the results across universities (Buckley et al. 2015; Maas 2009). Our results showed, of 18 universities that collected post-concussion symptom information, there was mixed use of the Graded Symptom Checklist (Janusz et al. 2012), Postconcussion Symptom Scale (Kontos et al. 2012; Lovell and Collins 1998; McCrea et al. 2003), and Concussion Symptom Inventory (Randolph et al. 2009). Although all of these scales have been validated among college athletes and cover most common symptoms associated with a sport-related concussion, the specific questions and the number of items on each scale varies (Kontos et al. 2012; McCrea et al. 2003; Randolph et al. 2009). For example, the Graded Symptom Checklist has 26 items (Janusz et al. 2012), while the Concussion Symptom Inventory has 12 items (Randolph et al. 2009). To allow for evidence-based, effective concussion management among collegiate athletes, efforts are needed to move towards standardized data collection including use of the same data collection tools across university athletic departments. A variety of co-sponsoring federal agencies participate in a scientific initiative to establish and refine TBI CDE standards (Grinnon et al. 2012; McCauley et al. 2012; Wilde et al. 2010), including the first set of CDEs for SRCs released in June 2017 (NINDS 2017). All these efforts will support a growing evidence base using CDEs while meeting the needs of each individual institution (Baugh and Kroshus 2016; Buckley et al. 2015; Kerr et al. 2015; Maas et al. 2011) and eventually inform practices allowing expansion towards data collection and monitoring in pre-collegiate levels.

Identifying the CDEs as the first step of data standardization not only facilitates collection of data and improves data quality, but also provides the foundation for data sharing and comparisons between studies. More than shared summaries of results, shared data, along with associated tools and methodologies, can help research advancement through re-analysis of data and/or re-aggregation and rigorous comparison with other data, methods, and tools. More recently, the Federal Interagency Traumatic Brain Injury Research informatics system was developed to provide a platform for data sharing across the entire TBI research field (Ivory 2015). The concussion CDEs identified through our study provide groundwork for a road map to common data definitions and standards, and subsequently to coherent informatics approaches for successful data sharing and results comparisons in concussion research among collegiate athletes (Maas et al. 2011; McCauley et al. 2012).

This study has several limitations. First, although over 90% of eligible universities voluntarily completed the survey, it is possible that non-participation was associated with low institutional effort on concussion data collection. Thus, our results might be biased towards universities that view CDEs in a more favorable light. Second, the data collected were based on the report of the Head ATs or Directors of Sports Medicine. While data were collected at the point of exposure and were part of injury monitoring, the results might reflect the participant’s perspective, or be subject to recall bias and/or participant bias. Third, the survey questions were largely focused on concussion management and safe return to play with little emphasis placed on the CDEs related to return to learn or effect of concussion on academic performance of student-athletes. Fourth, our study focused on current recommendations for standardized data collection elements. Information such as personal medical history, concussion specific comorbidity (i.e., history of headache, learning disability, psychiatric diagnoses) and advanced assessments (i.e., imaging, biomarkers, or genotyping) is valuable for better understanding concussion recovery but at this point not routinely collected. As evidence on the impact of these measures for athletic program decision-making advances, they are likely to become part of routine data collection. For example, these measures are included in current ongoing national study, Concussion Assessment, Research and Education (CARE) Consortium, on effects of concussion in collegiate athletes and US military service academy members (Broglio, et al., 2017). Finally, our study only included 19 Big Ten and Ivy League universities, which may be representative of large US universities, but unlikely to be representative of all universities.

## Conclusions

This study revealed important inter-institutional similarities and differences in concussion data collection in two large Division I conferences, with all universities collecting the recommended acute core CDEs but not many collecting in sub-acute or chronic timeframes. Based on the results obtained from this study, the authors have reached the following recommendations for future SRC studies among collegiate athletes that involve CDEs:

1. Collaborate across universities to collect as much standardized and comparable data as possible,
2. Collect more data on concussion specific premorbidity (e.g., history of headache, learning disabilities, and psychiatric diagnoses), and advanced assessments (e.g., neuroimaging, blood biomarkers, and genotyping),
3. Extend post-concussion evaluation beyond the acute period by including the sub-acute period (e.g., up to 3 months post-injury) and collect more standardized concussion outcome data including return to learn and academic performance,
4. Enable concussion data collected to be available for research by actively engaging IRB, researchers, and other research personnel before, during and after data collection, and
5. Translate current concussion data collection protocols and practices to smaller universities including Division 2 and 3 universities.

NCAA conferences or other affiliated institutional networks are uniquely positioned for collaborative research by establishing standardized data collection within the practical confines of athlete health care delivery. Leveraging such networks of institutions could allow for reliable identification of concussions using recommended CDEs, significant metadata results from data aggregation, and meaningful comparisons across studies or institutions, ultimately leading to strong evidence-based and individualized concussion prevention and treatment strategies unique to collegiate athletes.

## Abbreviations

AT: Athletic Trainers
CDE: Common data element
NCAA: National Collegiate Athletic Association
NINDS: National Institute of Neurological Disorders and Stroke
